# Delivering Palliative Care in Mental Health Nursing Settings: A Systematic Review

**DOI:** 10.1111/jpm.70115

**Published:** 2026-03-06

**Authors:** Oladapo Akinlotan, Allen O'Connor, Ruben Seetharamdoo, Mo Ghoorun

**Affiliations:** ^1^ Anglia Ruskin University Chelmsford UK; ^2^ Edge Hill University Ormskirk UK; ^3^ University of West London Brentford UK

## Abstract

**Rationale:**

Palliative care can provide comfort, alleviate suffering, and improve quality of life; however, access to palliative care for people with mental illnesses at the end of their lives is extremely poor. As the need for palliative care is expected to rise significantly in the future, palliative care must be considered a global health priority.

**Aim:**

To examine the provision of palliative care within mental health settings and explore the factors that influence the experience of patients receiving palliative care in these settings.

**Method:**

This systematic review draws on peer‐reviewed qualitative, quantitative and mixed‐methods primary studies, adhering to the Preferred Reporting Items for Systematic Reviews and Meta‐Analyses guidelines and was registered online. A total number of 61,782 studies was identified after a comprehensive search of five academic databases. After rigorous screening, only nine studies met inclusion criteria and were selected.

**Results:**

Thematic analysis identifies three major themes and three subthemes: access to palliative care, advance decisions and treatment, and care in palliative care settings (palliative care settings, palliative care professionals and palliative care/medical interventions).

**Conclusion:**

Access to palliative care for people with complex mental illness is very low when compared to the general population. Advance care planning should be initiated early in the development of palliative care needs, rather than at the point of mental illness diagnosis.

**Recommendations:**

Although care for people with complex mental illness is complex while dying, conversations around palliative care need to be as part of a therapeutic relationship and engagement. Also, palliative care staff have an important role in communicating end‐of‐life planning to patients' families and carers.

## Accessible Summary

What is known on the subject?

People with complex mental illness including those with dementia often have poorer access to palliative and end‐of‐life care compared with the general population. Barriers include lack of training for mental health staff in palliative care, stigma, and challenges in identifying physical health problems in psychiatric settings. Most previous research has focused on palliative care in general health or community settings rather than within mental health wards or hospitals.

What the paper adds to existing knowledge

This is the first systematic review to specifically examine how palliative care is delivered within mental health settings. The review synthesises findings from nine studies and identifies three key themes: access to palliative care, advance decision‐making, and treatment and care within palliative care settings. It highlights examples of good practice as well as gaps, including the need for better staff training, improved advance care planning, and more consistent communication with families.

What are the implications for practice?

Mental health nurses are likely to be increasingly involved in palliative care due to rising life expectancy and complex health needs amongst mental health service users. Training and support can help nurses manage physical symptoms, have sensitive conversations about care wishes, and work closely with families. Mental health services should be equipped to deliver compassionate, person‐centred palliative care that integrates both physical and psychological needs.

## 1 Introduction

Palliative care refers to interventions for patients at the end‐of‐life that aim to provide comfort, alleviate suffering and improve quality of life (World Health Organization (WHO) 2023). Palliative care focuses on alleviating symptoms of various chronic illnesses and is related to end‐of‐life or hospice care (Arnold 2022). For those with a mental illness, palliative care must also include psychological support and a broader conception of the concept of quality of life that reflects the complex realities of mental health; mental health nurses are uniquely positioned to provide this support (Masel et al. 2023). Palliative care has the potential to use an interdisciplinary approach to provide holistic care for patients with mental illnesses at the end of their lives (Masel et al. 2023). This can be achieved by improving the quality of life along the disease trajectory and to reduce symptom burden (Masel et al. 2023); however, the term ‘quality of life’ is a broad concept in the context of severe mental illness (Arnold 2022). Although often used interchangeably, end‐of‐life care focuses on support during the final months of life, addressing decline and terminal symptoms; whereas palliative care targets progressive, life‐threatening illnesses with no chance of remission, emphasising a holistic, interdisciplinary approach to maintain quality of life (Kim and Huh 2024; Van Mechelen et al. 2013).

As the global population is living longer, more people are dying at older ages with higher levels of chronic illness and dementia (Pask et al. 2025). Anxiety and depression are the most common mental health illnesses noted in older adults at the end‐of‐life (Masel et al. 2023), however people being cared for within mental health services frequently experience poorer access to palliative and end‐of‐life care, a finding widely documented across multiple reviews (Arnold 2022; Coffey et al. 2022; Wilson et al. 2020; Shalev et al. 2020). Millions of people die each year without access to palliative care (Peeler et al. 2024), as only one in ten who needs palliative care receives it (World Health Organisation 2021). As the need for palliative care is expected to increase significantly in the future (World Health Organisation 2021), palliative care must be considered a global health priority.

Palliative care is needed for people with a mental illness when comorbid health conditions reduce their life expectancy and lead to the need for end‐of‐life care (Blatt and Crawford 2015). Dementia is an exception in mental illnesses because it can lead to physical deterioration and reduce life expectancy (Connors et al. 2016). Despite making up about 4.5% of the global population (approximately 350 million), patients with a serious mental illness tend to have poor access to palliative care (Shalev et al. 2020). This is attributed in part to palliative care staff being ill equipped to support those with a serious mental illness (Edwards et al. 2021), and to the higher levels of chronic physical illness that are associated with their lifestyle choices (Sheridan 2019). Furthermore, the prevalence of dementia is expected to rise significantly in tandem with the aging population, with rates set to triple between 2019 and 2050 (Schwarzinger and Dufouil 2022). Stigma for dementia remains high, hindering timely access to appropriate care and support (Olwage 2024). Patients with dementia have poor access to hospice care (Lassell et al. 2022) and many clinicians have poor access to dementia‐specific training (Rasmussen et al. 2023). People with both functional and organic mental illnesses currently have a poorer experience of palliative care than the general population, and the demand for palliative support is likely to rise (Shalev et al. 2020). In this review, the term ‘complex mental illness’ is used to refer to long‐term, severe psychiatric conditions such as schizophrenia, bipolar disorder, and dementia. This differs from the term ‘serious mental illness’ because it also includes dementia.

This review has included both people with serious mental illness and dementia because both groups experience chronic, progressive cognitive or psychiatric impairment, experience barriers to accessing palliative care, and have overlapping holistic care needs (Erel et al. 2017; Lassell et al. 2022; Masel et al. 2023) and this will allow synthesis of insights across populations with significant mental health challenges. Furthermore, as this review is discussing the palliative care that takes place within mental health settings, the two populations will be receiving care in similar settings with comparable clinical teams including mental health nurses. Most of the previous systematic reviews into palliative care have focused their attention on outside of mental health settings (e.g., Coffey et al. 2022; Edwards et al. 2021). Miranda et al. (2019) discussed home‐based palliative care for dementia while Browne et al. (2021) reviewed how palliative care is defined, measured and understood. Riley et al. (2022) looked at how often palliative care is offered to people with a complex mental illness. Similarly, den Boer et al. (2019) conducted a systematic review of palliative care tools and interventions for people with severe mental illness, highlighting the lack of tailored approaches and the need for better integration of mental and physical health care. Unlike previous systematic reviews above, the aim of this current systematic review is to understand how palliative care is delivered in mental health nursing. The review aims to synthesise existing research relating to palliative care that is taking place within mental health settings and does not restrict findings to dementia or other serious mental illnesses unlike previous systematic reviews. For clarity, in this review the terms ‘advance directives’ and ‘advance decisions’ refers specifically to directives and decisions relating to palliative and end‐of‐life care. These terms can in other cases refer to decisions about mental health treatment and care. The research question is: how is palliative care delivered in mental health nursing?

## 2 Methodology

This study has chosen a systematic review method because systematic review provides a rigorous and transparent way to synthesise all relevant evidence and minimise bias (Egger et al. 2022). This systematic review explores palliative care in inpatient settings within a mental health nursing context, drawing on peer‐reviewed qualitative, quantitative, and mixed‐methods primary studies. It adheres to the PRISMA (Preferred Reporting Items for Systematic Reviews and Meta‐Analyses) guidelines (Page et al. 2021) and was registered with PROSPERO (CRD42024567248).

### 2.1 Search Strategy

A comprehensive literature search was conducted using CINAHL Complete, MEDLINE Complete, PsycINFO, APA PsycArticles, Psychology & Behavioural Sciences Collection. The search on the academic databases was supplemented with additional hand searches. All searches were conducted by two reviewers and were completed in August 2024. Search strategy used Boolean operators using the following search terms: Palliative OR ‘Palliative care’ OR ‘End‐of‐life care’ OR ‘End‐of‐life‐care’ OR ‘hospice care’ OR Dying OR death OR ‘terminal care’ OR ‘terminal treatment*’ OR ‘terminally ill’ AND ‘Psychiatr* nursing’ OR ‘mental health nursing’ OR Psychiatr* OR ‘mental health’ AND Ward* OR hospital* OR unit* OR facilit*.

### 2.2 Study Selection

**FIGURE 1:**
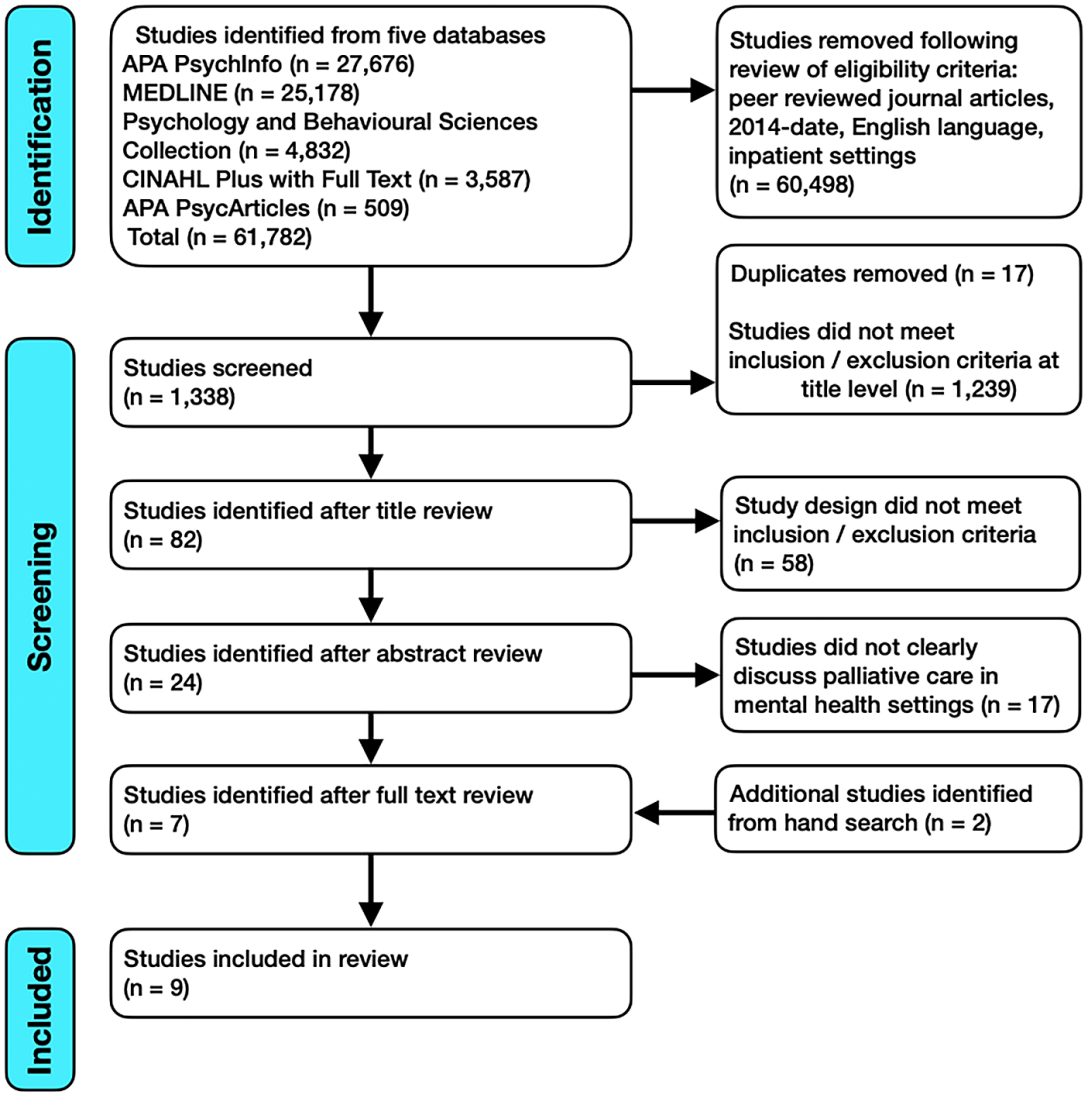
PRISMA flow diagram.

The search was limited to peer‐reviewed journal articles published in English from 2014 until 2024. A 10 year timeframe was applied from 2014 to 2024 to capture contemporary developments and exclude outdated models of care. An updated search covering 2024–2025 was completed in early September 2025 using the same databases, search terms and eligibility criteria but this yielded no additional eligible studies. Included studies focused on patients with a diagnosed mental health condition who were receiving treatment in inpatient settings and receiving palliative care during their admission. To be eligible for inclusion, study design must use qualitative, quantitative or mixed methods to examine the experience of patients receiving palliative care on mental health wards/settings (Table 1). A total of nine studies met the criteria and were selected for this review (Figure 1). Two reviewers conducted independent searches using the above databases and applied the eligibility criteria for possible inclusion of studies. Once the results were compared, a total number of 61,782 studies were identified for further screening. One reviewer conducted the initial screening including title review, and the second reviewer independently checked the identified studies. A total number of 23 studies were identified for full text review.

**TABLE 1:** The eligibility criteria for the studies considered.

	Inclusion	Exclusion
Study design and characteristics	Primary research studies. Peer reviewed journal articles. Studies published in English language. Studies published from 2014‐date	Articles that report no primary data. Secondary research, e. g. reviews. Studies published in non‐English language. Studies published prior to 2014.
Population/Participants	Studies that focus on psychiatric or mental health wards/units/facilities/hospitals. Patients may be of any age, gender, race or sexuality.	Studies that focus on psychiatric patients that are not on the wards. Studies that focus on non‐psychiatric wards.
Outcome	Studies that examined palliative care on psychiatric wards/units/facilities/hospitals. Qualitative studies that examined palliative care in psychiatric or mental health nursing. Quantitative studies that examined palliative care in psychiatric or mental health nursing. Studies that focus on palliative care in psychiatric or mental health wards/units/facilities/hospitals.	Studies that do not consider palliative care on psychiatric wards. Qualitative studies that do not examine palliative care in psychiatric or mental health nursing. Quantitative studies that do not examine palliative care in psychiatric or mental health nursing. Studies that considered other issues other than palliative care which is not presented separately.

### 2.3 Quality Assessment

The quality of the selected studies was assessed using the Mixed Methods Appraisal Tool (MMAT) tool (Table 2) for qualitative and quantitative research (Hong et al. 2018). This model was chosen because the MMAT is widely known for its content validity, and it has been applied successfully across diverse methodologies, including quantitative, qualitative, and mixed‐methods studies (Hutson and He 2024). All studies had clearly defined research questions and used appropriate data collection methods to address these questions (Table 2). The quantitative studies demonstrated good design features such as representative samples, suitable outcome measures, and consideration of confounding factors, indicating reliable internal validity (Table 2). Key appraisal criteria were also met by the qualitative studies as they used appropriate methodologies to examine complex psychosocial phenomena, showed consistency between data sources and interpretation and provided clear data references to support findings (Table 2). There were no significant methodological errors found, indicating that the results are reliable and relevant to palliative care for people with mental health needs (Table 2). Two reviewers independently completed full text review on the identified studies and after comparing results, a total number of nine studies were identified and agreed as the selected studies for the review. The characteristics of all included studies were extracted (Table 3).

**TABLE 2:** Quality appraisal of the included studies using mixed methods appraisal tool (MMAT) tool (Hong et al. 2018).

Quantitative studies							
	Are there clear research questions?	Do the collected data allow to address the research questions?	Are the participants representative of the target population?	Are measurements appropriate regarding both the outcome and intervention (or exposure)?	Are there complete outcome data?	Are the confounders accounted for in the design and analysis?	During the study period, is the intervention administered (or exposure occurred) as intended?
Ankuda et al. (2017)	Yes	Yes	Yes	Yes	Yes	Yes	Yes
Butler and O'Brien (2018)	Yes	Yes	Yes	Yes	Yes	Yes	Yes
Ding et al. (2020)	Yes	Yes	Yes	Yes	Yes	Yes	Yes
Epstein‐Lubow et al. (2015)	Yes	Yes	Yes	Yes	Yes	Yes	Yes
Hendriks et al. (2017)	Yes	Yes	Yes	Yes	Yes	Yes	Yes
Honinx et al. (2021)	Yes	Yes	Yes	Yes	Yes	Yes	Yes
Wilkins et al. 2019	Yes	Yes	Yes	Yes	Yes	Yes	Yes
**Qualitative studies**							
	Are there clear research questions?	Do the collected data allow to address the research questions?	Is the qualitative approach appropriate to answer the research question?	Are the qualitative data collection methods adequate to address the research question?	Are the findings adequately derived from the data?	Is the interpretation of results sufficiently substantiated by data?	Is there coherence between qualitative data sources, collection, analysis and interpretation?
Terpstra and Williamson 2019	Yes	Yes	Yes	Yes	Yes	Yes	Yes
Waterman et al. (2016)	Yes	Yes	Yes	Yes	Yes	Yes	Yes

**TABLE 3:** Summary of characteristics of included studies.

Author and Country	Aims	Study Design (SD) Participants Data Analysis (DA)	Key Findings	Limitations
Ankuda et al. (2017) United States	To examine hospice referral rates for hospitalised nursing home residents with advanced cognitive impairment (ACI) at the time of discharge between 2000 and 2010.	**SD**: Quantitative, retrospective cohort study. **Participants**: Hospitalised nursing home residents age ≥ 66 (*n* = 128,989). **DA**: Descriptive statistics. Multivariate Logistic regression.	Hospitalists were more likely than generalist physicians to refer patients to hospice care. Continuity of care was associated with lower hospice referral rates. Hospice referral rates increased from 2000 to 2010 for all physician types	The study showed an association between hospitalists and higher hospice referral rates but could not determine the underlying mechanisms for this association.
Butler and O'Brien (2018) New Zealand	To compare the rate of access to palliative care services for people with serious and persistent mental illness with the rate for the general population.	**SD:** Quantitative retrospective cohort study design. **Participants:** Adults diagnosed with Severe Mental Illness 18 years of age and over. **DA**: Descriptive statistics. Rate ratio.	People with serious and persistent mental illness (SPMI) are 3.5 times less likely to access specialist palliative care services compared to the general population.	The study did not account for palliative care provided within general health, mental health, or primary care services, only measuring specialist palliative care referrals.
Ding et al. (2020) Australia	To describe the clinical characteristics and symptoms of people diagnosed with dementia at the time of admission to inpatient palliative care.	**SD:** Quantitative, descriptive study. **Participants:** A total of 1872 patients with a primary diagnosis of dementia. **DA:** Descriptive statistics. Hierarchical Analysis.	People with dementia admitted to inpatient palliative care services had lower levels of symptom distress but higher functional impairment compared to those with cardiovascular disease, lung cancer, and motor neuron disease.	The assessment tools used were not specifically validated for dementia populations, which may have led to underestimation of care needs due to cognitive and communication difficulties.
Epstein‐Lubow et al. (2015) United States	To address the discharge disposition following inpatient psychiatric treatment for advanced dementia.	**SD:** Quantitative, secondary data analysis **Participants:** A population of 685, 305 patients with advanced cognitive and functional impairment, with a mean age of 85.9 years who had resided in a nursing home. **DA:** Descriptive analysis.	Only 8.7% individuals with advanced dementia in nursing homes were referred to hospice, with most discharged back to nursing homes without hospice services.	There is a lack of data on patient/family preferences and the views of healthcare providers.
Hendriks et al. (2017) Netherlands	To describe end‐of‐life treatment decisions for patients dying with dementia in various stages of dementia in long‐term care facilities in the Netherlands.	**SD:** Quantitative, secondary data analysis. **Participants:** A total of 330 residents with dementia who resided in 1 of 34 participating long‐term care facilities. **DA:** Descriptive statistics. T‐test and Fisher's exact test.	Physicians frequently withdrew life‐prolonging treatments such as artificial nutrition, hydration, oral medications and antibiotics shortly before death. Few residents had advance directives (4.9%), and hospitalisation rates in the last month of life were low (8%).	There was imprecise timing of decisions, combined retrospective and prospective data, and potential recall bias in retrospective assessments.
Honinx et al. (2021) Belgium, England, Finland, Italy, The Netherlands, and Poland	To estimate the prevalence of potentially inappropriate treatments (PIT) in the last week of life in nursing home residents and analyse the differences in prevalence between countries.	**SD:** Cross‐sectional study. **Participants:** A total of 1384 deceased residents from 322 nursing homes. **DA:** Descriptive statistics. Liner and Logistic Regression.	While the overall use of PITs was low, antibiotics were frequently administered, especially for residents with advanced dementia. In contrast, the prevalence of these treatments was significantly lower in some countries reflecting differences in palliative care approaches, cultural attitudes, and legal frameworks.	Reliance on nurse‐reported data, which may lead to recall bias, missing details on the clinical reasoning behind treatments, and the absence of information on when treatments were initiated.
Terpstra and Williamson 2019 United States	To describe the care of individuals who are terminally ill and diagnosed with borderline personality disorder.	**SD:** Qualitative, case studies, vignettes. **Participants:** A total of 3 terminally ill patients diagnosed with borderline personality disorder. **DA:** Discussion based on themes	Meet the challenge and build rapport. Be flexible and negotiate when possible. Recognise splitting behaviour and set boundaries.	It does not cover all of the issues that palliative care teams encounter when caring for terminally ill individuals with borderline personality disorder.
Waterman et al. (2016) United Kingdom	To illustrate difficulties of patients with dementia and life‐threatening comorbid conditions in receiving optimal end‐of‐life care.	**SD:** Qualitative, case study. **Participant:** A 55‐year‐old patient on the dementia ward at a psychiatric hospital. **DA:** Discussion based on themes	An effective end‐of‐life care can be provided in a psychiatric hospital, in accordance with recent new palliative care guidelines.	Study is limited to one patient on the dementia ward at a psychiatric hospital.
Wilkins et al. (2019) United States	To describe the characteristics of hospice referrals for people with dementia on discharge from inpatient geriatric psychiatry units.	**SD**: Retrospective chart review. **Participants**: A total of 133 inpatient admissions to the McLean Hospital Geriatric Cognitive Neuropsychiatry Unit between June 1, 2017, and July 15, 2018. **DA:** Descriptive statistics. Mean and standard deviation.	7% of patients were referred to hospice due to feeding problems. 78% of patients died within 31 days of discharge and 100% within 6 months. Feeding problems may indicate more rapid disease progression.	Difficulty with eating is seen as a sign of the dementia's progression, not a consequence of the patient's agitated state.

### 2.4 Data Synthesis

The findings from all included studies were extracted and compiled in a Word document and analysed using thematic analysis, following Braun and Clarke's (2006) model of thematic analysis. Thematic analysis has been chosen for this review because, while commonly applied to qualitative data, it can also be used to organise and interpret findings from quantitative studies, particularly when the aim is to draw out overarching themes rather than compare statistical outcomes (Kogen 2024; Braun and Clarke 2021). A quantitative meta‐analysis was not feasible due to the heterogeneity of study designs, populations, and outcome measures across the included studies. Instead, a thematic synthesis approach was used to integrate findings and identify key patterns relevant to palliative care delivery in mental health settings. Two reviewers independently conducted initial coding of the data. Their coding was then compared, and another independent reviewer carried out checks and descriptive coding. Descriptive codes were then checked independently by the three reviewers. Initial codes were grouped into descriptive codes, which were further refined into emerging themes. Recurring and common themes across studies were identified and analysed to determine the key overarching themes. All four reviewers participated in the process of thematic analysis. One reviewer conducted the initial identification of the major themes, and these were independently checked by the other three reviewers. Any disagreements and discrepancies were resolved through comparison of their initial coding and additional discussions.

## 3 Results

Thematic analysis identifies three major themes: access to palliative care, advance decisions, and treatment and care in palliative care settings. Treatment and care in palliative care settings has three subthemes (palliative care settings, palliative care professionals, and palliative care/medical interventions).

### 3.1 Access to Palliative Care

Seven out of nine studies (Butler and O'Brien 2018; Ding et al. 2020; Waterman et al. 2016; Wilkins et al. 2019; Epstein‐Lubow et al. 2015; Ankuda et al. 2017; Terpstra and Williamson 2019) discussed issues around access to palliative care which include access to palliative care services, referral, and admission to palliative care settings. When compared to the general population, access to palliative care for people with mental illness is very low (Butler and O'Brien 2018; Ding et al. 2020). For example, the rate of access to palliative care is 0.5% for the complex mental illness population in New Zealand while the rate of access for the general population is 1.72% (Butler and O'Brien 2018). Similarly, those with complex mental illness are 3.5 times less likely to access specialist palliative care in New Zealand (Butler and O'Brien 2018). In Canada, people with schizophrenia are 2–3 times less likely to access specialist inpatient palliative care (Butler and O'Brien 2018).

Access to inpatient palliative services need to be prioritised for people with dementia while equitable access should be emphasised for palliative care (Ding et al. 2020). For people with complex mental illness stigma, poor awareness, high level of deprivation and health professionals' perspectives of mental illness are common barriers preventing access to specialist palliative care (Butler and O'Brien 2018). As people are living longer with dementia, it will become more important for a wider range of services to cater for palliative care needs of dementia patients as these will come with a heightened financial cost due to complexity of care (Waterman et al. 2016). The increase in people dying with dementia and complexity in co‐morbidities impacts numbers of people with dementia accessing specialist palliative care (Ding et al. 2020). Similarly, complexities relating to dementia act as a barrier to accessing hospice care (Waterman et al. 2016); however, more research into hospice eligibility for dementia patients is required (Wilkins et al. 2019).

There is a low rate (7%–9%) of referral to hospice (Wilkins et al. 2019; Epstein‐Lubow et al. 2015). On the other hand, there was a significant rise in hospice referrals between 2000 and 2010 with advanced dementia in New Hampshire USA and there is evidence that speciality: generalist or specialist impacts referrals (Ankuda et al. 2017). Reasons for higher rates of dementia referrals to hospice care include: speciality, high number of patients, improved prognostication, reduced fears of patients feeling abandoned and financial incentives to reduce admissions and inpatient deaths (Ankuda et al. 2017). On the other hand, lack of referrals are caused by lack of appropriate services, misconceptions of palliative care, difference in prognostication and referral being seen as disruption (Ankuda et al. 2017).

When an individual with advanced dementia is admitted into an inpatient psychiatric care, it is important to explore if the person may be near end‐of‐life care (Epstein‐Lubow et al. 2015). Suitable methods should be used to evaluate an individual with advanced dementia for their eligibility for hospice while family caregivers should be assured in discussions for hospice (Epstein‐Lubow et al. 2015). Patients with Borderline Personality Disorder (BPD) with complex mental illness have challenges for admission to inpatient palliative care settings (Terpstra and Williamson 2019).

### 3.2 Advance Decision Making

Three out of nine studies (Butler and O'Brien 2018; Hendriks et al. 2017; Waterman et al. 2016) discussed issues around advance decisions in palliative care which include advance decision making, advance directives, capacity and competence, and best interest decisions. Specialist planning for palliative care should take place as soon as dementia is diagnosed; however, a major barrier to advance decisions is that dementia may not be identified early (Waterman et al. 2016). This is more important because most nursing home staff and families do not consider dementia a terminal illness and this necessitates vital education about dementia (Waterman et al. 2016). The importance of early advance decisions being made while a person still has capacity has been emphasised (Waterman et al. 2016). A complex mental illness population needs to have better access to advance care planning, and this will allow professionals to negotiate future care (Butler and O'Brien 2018).

In Europe, most patients do not have written advance directives at the point of admission to long term care facilities while a very small number of nursing home residents with dementia have an agreed advance directive (Hendriks et al. 2017). Compared to Europe, in the USA, written advance directives are popular as a greater level of directives is reported in older people on the day of death (Hendriks et al. 2017). The numbers of advance directives are possibly influenced by culture and type of organisational models (Hendriks et al. 2017).

Patients with reduced advanced dementia, had decision‐making competence of a greater level than those with advanced dementia in the last week of life (Hendriks et al. 2017). Generally, an advance directive includes a lot of decisions including but not limited to treatment, medications, resuscitation, artificial nutrition and hydration and where to die (Hendriks et al. 2017; Waterman et al. 2016). A minority of patients had a level of competence for decision making regarding medical intervention during admission, but this reduced by over half in the final week of life (Hendriks et al. 2017). When patients no longer have capacity or competence in decision‐making, most discussions regarding end‐of‐life treatment decisions normally take place with proxy decision makers (Hendriks et al. 2017). In this case, family will play an active role in best interest decision making alongside (MDT) multidisciplinary team (Waterman et al. 2016; Hendriks et al. 2017).

### 3.3 Treatment and Care in Palliative Care

Eight out of nine studies (Butler and O'Brien 2018; Ding et al. 2020; Waterman et al. 2016; Wilkins et al. 2019; Epstein‐Lubow et al. 2015; Terpstra and Williamson 2019; Honinx et al. 2021; Hendriks et al. 2017) discussed issues around access to treatment and care in palliative care under three subthemes: palliative care settings, palliative care professionals and palliative care interventions.

#### 3.3.1 Palliative Care Settings

Nursing home patients with advanced dementia are a vulnerable population (Epstein‐Lubow et al. 2015). Similarly, patients admitted into impatient palliative care services with dementia have prolonged functional decline, substantial functional impairment, increased impairment and disability (Ding et al. 2020). These patients require greater support with activities of daily living compared to patients diagnosed with cancer, organ failure or other conditions (Ding et al. 2020). This suggests that patients with dementia being older, are most likely to be admitted to impatient services at a deteriorating or terminal phase including lower performance levels and shorter admission prior to death (Ding et al. 2020).

People with advanced dementia accessing palliative care services requires treatment in a familiar setting, for reducing risk of biopsychological distress because of changes in everyday routine (Ding et al. 2020). Similarly care amongst people with dementia requires assurance of appropriateness of setting where palliative management is best provided (Ding et al. 2020) while continuity of care is vital for optimal palliative care (Waterman et al. 2016). Epstein‐Lobow et al. (2015) reported that there was a transition to at least one new treatment environment in the last year of life for most individuals studied (Epstein‐Lobow et al. 2015). However inpatient transfers can escalate risk of complications and can lead to reduced quality of life, and increased mortality risk (Ding et al. 2020). A risk assessment is required to ensure advantages of inpatient care are balanced against an unknown environment (Ding et al. 2020). Inpatient setting can include support and resources such as spiritual support from chaplaincy which are not always available in other clinical settings (Epstein‐Lubow et al. 2015; Waterman et al. 2016).

Palliative care can be provided in a mental health setting, where patients are familiar with, have the freedom to move around the ward, have continuity of care and where their psychosocial needs can be addressed (Waterman et al. 2016). Deficits in staff competency can be filled by liaising with palliative teams while team members may benefit from specific end‐of‐life guidance and training. (Waterman et al. 2016). Hence it is important for mental health trusts to formulate end‐of‐life care policies for in‐patient settings and these policies should be informed by care homes/palliative care centres (Waterman et al. 2016).

#### 3.3.2 Palliative Care Professionals

The experience of palliative care staff to recognise signs of end‐of‐life is useful to communicate this to family, plan and update MDT‐based comprehensive care plans (Waterman et al. 2016). Hospice care for people with dementia can be challenging due to uncertainty of prognosis and difficulty in prognosis which lead to a wider range of hospice admission lengths (Wilkins et al. 2019). Consequently, prognostic estimates can be less helpful when determining hospice eligibility (Wilkins et al. 2019). Having a greater appreciation of acute psychiatric symptoms over the span of advancing dementia will assist in efforts for improving the quality of palliative care for persons with severe cognitive impairment (Epstein‐Lubow et al. 2015). Healthcare professionals need support to monitor impacts of the symptoms of residents with dementia to allow seamless intervention for symptom management (Ding et al. 2020). Dementia patients that exhibit behavioural symptoms may be in a more rapid decline (Wilkins et al. 2019).

Whilst most inpatient clinicians have skills in meeting the holistic needs of patients, they have limitations in managing borderline personality disorder (BPD) symptoms, and disagreements amongst clinicians may lead to inadequate care (Terpstra and Williamson 2019). Using palliative psychology, effective team working, having a written care plan, and adopting standard treatment options for care settings to manage differing approaches with individuals with BPD are useful recommendations (Terpstra and Williamson 2019). With an increasing older population with advanced dementia, the need for specialist palliative care clinicians with appropriate skill mixes is essential, but limitations on the workforce for specialist palliative care are a challenge globally (Ding et al. 2020). Collaboration between professionals is important to improve palliative care for people with complex mental illness, as health professionals can have a positive effect and influence (Butler and O'Brien 2018).

#### 3.3.3 Palliative Care Interventions

There are lower levels of symptom distress and functional ability for people with dementia in comparison with the other conditions (Ding et al. 2020). It has been noted that nurses frequently underrate symptoms of dementia in comparison with patient rating (palliative care screening) and this may have led to underestimating symptom distress (Ding et al. 2020). The system for palliative care screening does not differentiate people being admitted with a diagnosis of dementia from those with co‐morbidities and other conditions (Ding et al. 2020).

Some patients on palliative admissions sometimes have primary diagnoses with a range of physical illnesses including but not limited to cardio‐vascular disease (CVD), stroke, cancer or motor neurone disease (Ding et al. 2020). It has been noted that poor training causes mental health professionals to be reluctant to access physical health care needs for patients with complex mental illness populations (Butler and O'Brien 2018). Also, separation of physical and mental health services creates barriers (Butler and O'Brien 2018). Breathing problems are more common than other symptoms (Ding et al. 2020) while critical care interventions such as the use of artificial ventilation vary from one country to another (Honinx et al. 2021). Other patients have feeding problems with food and fluid intakes that are insufficient to sustain life (Wilkins et al. 2019). For some patients with weight loss issues, appetite‐stimulating medications are normally prescribed (Wilkins et al. 2019). Other patients are treated with rehydration therapy due to conditions such as dehydration and pneumonia (Hendriks et al. 2017).

Artificial Nutrition and/or hydration such as tube feeding is considered a form of treatment for reasons due to vomiting because of cerebrovascular accident, impact of sepsis or due to minimal conscious state (Hendriks et al. 2017; Honinx et al. 2021). The decision for artificial Nutrition and/or hydration should be considered during advance care planning (Waterman et al. 2016). It is common practice not to start with artificial nutrition and hydration in patients with dementia within the nursing home setting due to physicians accepting reduction in foods and fluid intake as part of the dying process (Hendriks et al. 2017). Other treatment options include blood transfusion, chemotherapy, radiotherapy, dialysis and surgery (Honinx et al. 2021). The use of clinical/medical interventions in the last week of life is rare and there is no use of chemotherapy or radiotherapy in the final week of life across different countries (Honinx et al. 2021).

Regarding pharmacological intervention, antibiotics are commonly prescribed treatment for pneumonia, urinary‐tract infections, skin infections and other conditions in the final week of life (Hendriks et al. 2017; Honinx et al. 2021). Decisions on treatment do not vary between patients with advanced and reduced dementia; however, patients with less advanced dementia are prescribed antibiotics more frequently than those with advanced dementia (Hendriks et al. 2017). It remains unclear if antibiotics enhance comfort, but their likely benefits need to be weighed against the risk of possible adverse effects including prolonging the dying process and resistance to antibiotics (Hendriks et al. 2017). Other medications used with varying degrees across different countries are antidiabetics, oral anti‐coagulants and statins (Honinx et al. 2021). The use of potentially inappropriate treatment in the last week of life with varying degrees across countries has been reported (Honinx et al. 2021).

Although care for people with complex mental illness is laid with complexity while dying, conversations around palliative care need to be as part of a therapeutic relationship (Butler and O'Brien 2018). Whenever life‐prolonging medical intervention is considered, quality of life should also form an important part of the decision process for end‐of‐life care (Hendriks et al. 2017). Where treatment was withdrawn, the type of treatments often withdrawn included all oral medication, antibiotics, and other drugs including hydration or tube feeding (Hendriks et al. 2017). Health professionals frequently face ethical dilemmas regarding commencing and ending treatment, especially when this does not add to the quality of life (Hendriks et al. 2017). Common causes of death are severe cardiovascular disease and cancer, while mean age at death is between 81 in Poland and 87 in Belgium and England (Honinx et al. 2021). The route to dying varies and includes euthanasia, withdrawal of treatment, lack of resuscitation, and natural causes (Hendriks et al. 2017).

## 4 Discussion

This is the first systematic review to specifically examine the provision of palliative care within mental health settings. Findings from this review highlight several factors that influence the experience of patients receiving palliative care in mental health inpatient settings. These include access to palliative care, advance decision making, and the treatment and care that patients receive when they are in mental health inpatient hospitals or settings (e.g., Butler and O'Brien 2018; Hendriks et al. 2017; Waterman et al. 2016; Wilkins et al. 2019; Ding et al. 2020).

Patients with a complex mental illness have poor access to palliative care when compared to the general population (Butler and O'Brien 2018; Ding et al. 2020). The wider literature also highlights this finding, attributing diagnostic overshadowing (Irwin et al. 2014; Hallyburton 2022) and the stigma associated with complex mental illness (Gerhart et al. 2023) as major causes (Wilson et al. 2020). Patients with a diagnosis of BPD present additional challenges for palliative care settings (Terpstra and Williamson 2019). Feely et al. (2013) suggest that patients with a personality disorder can feel exaggerated degrees of suffering at the end of life, and that teams often lack the skills to support them. This lack of access to palliative care also extends to a lower rate of hospice referrals for patients with dementia already receiving mental health support (Wilkins et al. 2019; Epstein‐Lubow et al. 2015), in part due to the heightened cost of care (Waterman et al. 2016) and the experience of the doctor assessing the patient (Ankuda et al. 2017). In a study of hospice interventions for patients with dementia, Lassell et al. (2022) recognise significant inequalities in accessing hospice settings compared to the general population. Healthcare professionals often lack dementia‐specific training, which acts as a barrier to admission to certain clinical settings (Erel et al. 2017; Rasmussen et al. 2023). These findings echo previous research highlighting staff‐reported barriers to providing palliative care for people with severe mental illness, including limited training and organisational constraints (Jerwood et al. 2018). In addition, Jerwood et al. (2021) highlight the perspectives of patients and carers themselves, reporting that people with severe mental illness often experience fragmented communication, uncertainty about care pathways, and a sense of professionals ‘stepping back’ at the end of life. Their findings reinforce the need for proactive, relational approaches to palliative care within mental health services and support the themes identified in this review.

Most mental health patients in Europe do not have advance directives or advance decisions in place but this is less common in the USA potentially due to the difference in healthcare systems (Hendriks et al. 2017). Conversation around palliative care for people with a complex mental illness needs to be part of a therapeutic relationship, including discussions around quality of life and advance decision making (Butler and O'Brien 2018; Hendriks et al. 2017). In the UK this has led to a suggested reform of mental health legislation with a greater focus on advance decision making (Owen et al. 2019), though this is yet to come into force. Poor detection of dementia can be a barrier to putting an advance decision in place while patients still have the capacity to do so (Waterman et al. 2016), although patients with psychosis and mood disorders tend to have better access to advance decision making and consequently have better outcomes (Butler and O'Brien 2018). Treatment that includes artificial nutrition, medication, and hydration should be considered during advance decision making, which can be problematic for patients with advanced dementia (Hendriks et al. 2017; Honinx et al. 2021; Waterman et al. 2016). Kinch et al. (2024) suggest that the use of advance directives is limited in dementia patients, with patients often having little input into the planning. van Keijzer‐Laarhoven et al. (2020) add that healthcare staff engaging with advance care planning for dementia patients often face complex ethical problems that delay decision making.

People with dementia accessing palliative care require familiarity and continuity, often resulting in clinicians weighing up the value of a move to a palliative setting (Ding et al. 2020; Waterman et al. 2016), consequently mental health settings should be equipped to provide palliative care (Waterman et al. 2016). Patients with advanced dementia are a vulnerable group that require a high level of complex support (Epstein‐Lubow et al. 2015; Ding et al. 2020). Ryman et al. (2019) state that relocation for patients with dementia can have significant negative effects on their physical and psychological health. There is a likelihood that key information about a dementia patient and their care can be lost during a transition which can negatively impact continuity of care (Ashbourne et al. 2021). It has been suggested that dementia specific environments are tailored to the complex needs associated with the illness, making them the most effective places to address the psychological wellbeing of these patients (Quirke et al. 2023; Fleming et al. 2015).

Palliative care staff have an important role in communicating end‐of‐life planning to a patient's family (Waterman et al. 2016). Research has found that such support meetings do not have a significant impact on family anxiety and depression (Nowels et al. 2023; Carson et al. 2016); however, families can play a key role in planning and advance decision making for their loved ones (Kinch et al. 2024). Palliative care staff must have an appreciation of acute psychiatric conditions to improve the quality of palliative care (Epstein‐Lubow et al. 2015; Ding et al. 2020; Wilkins et al. 2019). Patients with BPD may receive inadequate end‐of‐life care due to clinicians not having the skills to manage their needs (Terpstra and Williamson 2019). This can prove difficult when care is provided by nurses who are not specifically trained in mental health, as nurses from other fields may have gaps in their mental health knowledge (McInnes et al. 2022; Ryan et al. 2021). An increasingly older population will have more people experiencing advanced dementia, so the need for more specialist palliative care is vital (Ding et al. 2020; Butler and O'Brien 2018). According to the World Health Organisation (WHO) (2021), global demand for palliative care is currently about 57 million, but this is set to double to nearly 114 million by 2060. However, current levels of knowledge around palliative care are low within the field of mental health nursing (Liu et al. 2025; Coffey et al. 2022), highlighting the urgent need for well‐trained palliative care professionals.

It is more difficult to assess the distress caused by physical health problems in patients with dementia due to the nature of their presentation (Ding et al. 2020). Diagnostic overshadowing is a problem faced by many people with a mental illness (Liberati et al. 2025), but this problem is complicated further by issues surrounding communication and capacity relating to advanced dementia (Bunk et al. 2021; Achterberg et al. 2019). Staff‐reported barriers are well documented, with multiple reviews describing inadequate training, poor integration of physical and mental health care, and uncertainty about managing complex comorbidities (Coffey et al. 2022; Edwards et al. 2021; Jabbie et al. 2024; McInnes et al. 2022). There is a recognition that some mental health nurses lack physical health knowledge and practical skills (Jabbie et al. 2024; Dorey et al. 2023). Knippenberg et al. (2023) found that people with severe mental illness often felt their physical symptoms were overlooked, and they experienced uncertainty and anxiety about how their mental and physical health needs would be balanced during palliative care.

Common physical health symptoms encountered during palliative care for those with a mental illness include breathing difficulties requiring treatments such as ventilation (Ding et al. 2020; Honinx et al. 2021), and difficulties taking in food and fluids requiring the use of artificial feeding/hydration (Wilkins et al. 2019; Hendriks et al. 2017). Breathing difficulties are common in people experiencing palliative care and require a holistic approach that includes treating a person's emotional state (Crombeen and Lilly 2020), as anxiety can further exacerbate breathlessness. Palliative interventions for breathlessness improve a patient's quality of life (Santos and Reis‐Pina 2023). This may be more important for patients who are already experiencing anxiety as part of their mental health presentation. Artificial feeding and hydration can improve quality of life by alleviating thirst and can prevent opioid toxicity; however, it can also worsen nausea symptoms and put patients at risk of aspiration (Carter 2020). Despite recognising its improvement to quality of life, many clinicians oppose the use of artificial nutrition viewing it as an aggressive treatment for palliative care (Pala et al. 2025).

## 5 Strengths and Limitations

This is the first systematic review to focus specifically on the delivery of palliative care within mental health nursing settings, addressing a major gap in the literature. The review follows a rigorous methodology, including PROSPERO registration, adherence to PRISMA guidelines (Page et al. 2021), and quality appraisal using the Mixed Methods Appraisal Tool (Hong et al. 2018) to ensure transparency and reliability. Findings provide critical insights into barriers and enablers of palliative care in mental health contexts, offering evidence to guide clinical practice, inform workforce training, and shape service development to improve outcomes for this highly vulnerable population. This review has several limitations that should be considered when interpreting the findings. Only nine studies met the inclusion criteria, which does not necessarily limit the discussion but does represent a lack of studies in this topic. A great number of studies may have allowed for greater generalisability. Most studies were conducted in high‐income countries, which may limit applicability to low‐ and middle‐income settings where mental health and palliative care resources differ significantly. Finally, there is a risk of publication bias, as unpublished studies and grey literature were not included; however, we have limited included studies to peer‐reviewed studies to ensure high level of rigour and quality is maintained.

## 6 Conclusion

This is the first systematic review to specifically examine the provision of palliative care within mental health settings. Drawing on peer‐reviewed qualitative, quantitative and mixed‐methods primary studies identified from relevant academic databases and adhering to the PRISMA guidelines, the study examines the provision of palliative care within mental health settings and explores the factors that influence the experience of patients receiving palliative care in mental health inpatient settings. Thematic analysis identifies three major themes: access to palliative care, advance decisions and treatment, and care in palliative care settings while treatment and care in palliative care settings has three subthemes (palliative care settings, palliative care professionals and palliative care interventions). When compared to the general population, access to palliative care for people with mental illness is very low. Specialist planning for palliative care should take place as soon as any complex mental illness such as dementia is diagnosed. In Europe, most patients do not have written advance directives at the point of admission to long‐term care facilities. The use of clinical/medical interventions in the last week of life is rare and there is no use of chemotherapy or radiotherapy in the final week of life across different countries. People with dementia accessing palliative care require familiarity and continuity; hence, mental health settings should be equipped to provide palliative care. Care for people with complex mental illness is generally complex while dying; however, conversations around palliative care need to be as part of a therapeutic relationship. Similarly, palliative care staff have an important role in communicating end‐of‐life planning to patients' families, relatives and caregivers.

## 7 Relevance to Mental Health Nursing

People with complex mental illnesses experience significant barriers to accessing palliative care, leading to poorer outcomes at the end of life and higher rates of chronic illness. Mental health nurses play a crucial role in coordinating and delivering care in these settings, yet little is known about best practices for integrating palliative care within mental health services. This systematic review synthesises evidence on access, decision‐making, and care delivery, highlighting gaps in training, policy, and practice. Findings can inform service development, guide nurse education and support mental health nurses to provide compassionate, person‐centred end‐of‐life care.

## Conflicts of Interest

The authors declare no conflicts of interest.

## Data Availability

Data sharing not applicable to this article as no datasets were generated or analysed during the current study.
